# Colvara BC, Ritzel IF, Aguiar VR, Hilgert JB, Celeste RK. Cobertura do Programa Bolsa Família e fatores associados à realização de procedimentos odontológicos no Brasil, entre 2007 e 2011: um estudo ecológico. Cad Saúde Pública 2023; 39(7):e00200622.

**DOI:** 10.1590/0102-311XER200622

**Published:** 2023-10-09

Onde se lê:

**Tabela 2:** Modelos finais analisando o aumento na taxa de quatro categorias de procedimentos odontológicos e a taxa de cobertura do Programa Bolsa Família, das Estratégias Saúde da Família (ESF) e das equipes de saúde bucal (EqSB) entre os períodos de 2007/2008 e 2010/2011 nos 5.570 municípios brasileiros.

	Aumento de procedimentos coletivos *		Aumento de procedimentos preventivos **		Aumento de procedimentos restauradores ***		Aumento de procedimentos exodônticos ^#^	
	OR	IC95%	OR	IC95%	OR	IC95%	OR	IC95%
Cobertura do Programa Bolsa Família								
Aumentou > 40p.p.	1,21	0,99-1,47	1,49	1,23-1,80				
Aumentou até 20-40p.p.	1,12	0,95-1,31	1,12	0,95-1,31				
Aumentou até 20p.p.	1,00		1,00		1,00		1,00	
Reduziu	1,12	0,95-1,30	1,18	1,02-1,38	1,01	0,89-1,17	0,98	0,83-1,15
Cobertura das EqSB								
Aumentou	1,14	0,98-1,33	1,41	1,20-1,61	1,75	1,51-2,04	1,41	1,20-1,63
Manteve	1,09	0,93-1,28	1,14	0,97-1,34	1,28	1,09-1,51	1,03	0,87-1,22
Reduziu	1,00		1,00		1,00		1,00	
Cobertura das ESF								
Aumentou			1,05	0,94-1,17	0,97	0,86-1,08		
Manteve			1,31	0,96-1,80	1,40	1,02-1,91		
Reduziu			1,00		1,00			

p.p.: pontos percentuais.

Modelos finais ajustados para:

* Cobertura inicial do Bolsa Família, porcentagem de mulheres, idosos e crianças de até 14 anos;

** Cobertura inicial do Bolsa Família, porcentagem da população em extrema pobreza e renda *per capita* média;

*** Porte populacional do município, porcentagem de crianças de até 14 anos e renda *per capita* média;

^#^ Cobertura inicial do Bolsa Família, porte populacional do município, porcentagem de mulheres, crianças de até 14 anos e idosos.

Leia-se:

**Tabela 2:** Modelos finais analisando o aumento na taxa de quatro categorias de procedimentos odontológicos e a taxa de cobertura do Programa Bolsa Família, das Estratégias Saúde da Família (ESF) e das equipes de saúde bucal (EqSB) entre os períodos de 2007/2008 e 2010/2011 nos 5.570 municípios brasileiros.

	Aumento de procedimentos coletivos *		Aumento de procedimentos preventivos **		Aumento de procedimentos restauradores ***		Aumento de procedimentos exodônticos ^#^	
	OR	IC95%	OR	IC95%	OR	IC95%	OR	IC95%
Cobertura do programa Bolsa Família								
Aumentou > 40p.p.	1,21	0,99-1,47	1,49	1,23-1,80	1,46	1,25-1,70	1,14	0,93-1,38
Aumentou entre 20-40p.p.	1,12	0,95-1,31	1,12	0,95-1,31	1,11	0,96-1,27	0,87	0,74-1,03
Aumentou até 20p.p.	1,00		1,00		1,00		1,00	
Reduziu	1,12	0,95-1,30	1,18	1,02-1,38	1,01	0,89-1,17	0,98	0,83-1,15
Cobertura das EqSB								
Aumentou	1,14	0,98-1,33	1,41	1,20-1,61	1,75	1,51-2,04	1,41	1,20-1,63
Manteve	1,09	0,93-1,28	1,14	0,97-1,34	1,28	1,09-1,51	1,03	0,87-1,22
Reduziu	1,00		1,00		1,00		1,00	
Cobertura das ESF								
Aumentou			1,05	0,94-1,17	0,97	0,86-1,08		
Manteve			1,31	0,96-1,80	1,40	1,02-1,91		
Reduziu			1,00		1,00			

IC95%: intervalo de 95% de confiança; OR: *odds ratio*; p.p.: pontos percentuais.

Modelos finais ajustados para:

* Cobertura inicial do Bolsa Família, porcentagem de mulheres, idosos e crianças de até 14 anos;

** Cobertura inicial do Bolsa Família, porcentagem da população em extrema pobreza e renda *per capita* média;

*** Porte populacional do município, porcentagem de crianças de até 14 anos e renda *per capita* média;

^#^ Cobertura inicial do Bolsa Família, porte populacional do município, porcentagem de mulheres, crianças de até 14 anos e idosos.

